# Cortical parvalbumin and somatostatin GABA neurons express distinct endogenous modulators of nicotinic acetylcholine receptors

**DOI:** 10.1186/s13041-014-0075-9

**Published:** 2014-10-31

**Authors:** Michael P Demars, Hirofumi Morishita

**Affiliations:** Department of Psychiatry, Icahn School of Medicine at Mount Sinai, One Gustave L. Levy Place, Box 1230, New York, NY 10029 USA; Department of Neuroscience, Icahn School of Medicine at Mount Sinai, One Gustave L. Levy Place, New York, NY 10029 USA; Department of Ophthalmology, Icahn School of Medicine at Mount Sinai, One Gustave L. Levy Place, New York, NY 10029 USA; Mindich Child Health and Development Institute, Icahn School of Medicine at Mount Sinai, One Gustave L. Levy Place, New York, NY 10029 USA; Friedman Brain Institute, Icahn School of Medicine at Mount Sinai, One Gustave L. Levy Place, New York, NY 10029 USA

**Keywords:** GABA, Parvalbumin, Somatostatin, Lynx family, Lynx1, Lypd6, Nicotinic acetylcholine receptor, Visual cortex, Mouse

## Abstract

**Background:**

Inhibition from GABAergic interneurons in brain circuits is a critical component of cognitive function. This inhibition is regulated through a diverse network of neuromodulation. A number of recent studies suggest that one of the major regulators of interneuron function is nicotinic acetylcholinergic transmission and dysregulation of both systems is common in psychiatric conditions. However, how nicotinic modulation impacts specific subpopulations of diverse GABAergic interneurons remains in question. One potential way of conferring specificity to the convergence of GABAergic and nicotinic signaling is through the expression of a unique family of nicotinic acetycholine receptor modulators, the Lynx family. The present study sought to identify members of the Lynx family enriched in cortical interneurons and to elucidate subpopulations of GABAergic neurons that express unique nicotinic modulators.

**Results:**

We utilize double fluorescence *in situ* hybridization to examine the interneuronal expression of the Lynx family in adult mouse visual cortex. We find that two of the Lynx family members, Lynx1 and Lypd6, are enriched in interneuron populations in cortex. Nearly all parvalbumin interneurons express Lynx1 but we did not detect Lypd6 in this population. Conversely, in somatostatin interneurons Lypd6 was found in a subset localized to deep cortical layers but no somatostatin neurons show detectable levels of Lynx1. Using a combination of genetic and viral manipulations we further show that a subpopulation of deep-layer cortico-cortical long-range somatostatin neurons also express Lypd6.

**Conclusions:**

This work shows that distinct subpopulations of GABAergic interneurons express unique Lynx family members. The pattern of expression of Lynx family members within interneurons places them in a unique position to potentially regulate the convergence of GABAergic and nicotinic systems, dysfunction of which are characteristic of psychiatric disorders.

**Electronic supplementary material:**

The online version of this article (doi:10.1186/s13041-014-0075-9) contains supplementary material, which is available to authorized users.

## Background

GABAergic interneurons provide the major source of inhibition to cortical networks. The proper functioning of GABAergic circuits are critical for appropriate network activity as animal models of dysfunctional GABAergic signaling show deficits in cortical plasticity [1-5], a disruption of normal synchronous oscillations [6,7] and cognitive deficits [6,8]; common hallmarks associated with psychiatric disorders such as Schizophrenia [6,9,10]. GABAergic interneurons, however, are an extremely diverse population that can be molecularly classified into three non-overlapping groups based on the expression of either Parvalbumin (PV), Somatostatin (SST) or serotonin receptor 3a (5-HT_3A_R) that together encompass nearly 100% of all cortical interneurons [11]. Particularly, deficits in both PV and SST interneurons have been reported in the brains of Schizophrenic patients upon post-mortem analysis [12-14], and both PV and SST neurons have been implicated in governing network oscillations in the brain [6,15]. Thus the precise modulation of distinct interneuron populations could have a significant impact on brain function and disease.

One of the major sources of neuromodulatory activity onto GABAergic neurons is through nicotinic acetylcholinergic signaling. Nicotinic signaling is pivotal to a number of cognitive processes including attention and learning and memory processes and is thought to contribute to synchronous oscillatory activity [16-20]. Indeed, developmental nicotinic dysfunction may contribute to neuropsychiatric conditions including schizophrenia [21]. In the neocortex, interneurons are the major target of basal forebrain derived nicotinic signaling [22-24]. Among GABAergic subtypes, this signaling is divergent, differentially expressed across cortical lamina and driven through varying nicotinic acetylcholine receptor (nAChR) subunits [25,26]. However, the manner in which tight regulation of GABAergic-nicotinic convergence is achieved at the interneuronal subtype level remains to be elucidated.

The recent discovery of the Lynx family provides a potential new layer of modulation of nicotinic signaling. The Lynx family is a member of the Ly-6/uPAR superfamily which is characterized by a three-looped folding structure or toxin fold due to a similarity to snake venom toxins such as α-bungarotoxin. These toxin-like proteins can bind to the extracellular face of nAChRs and ultimately augment signaling [27]. Recent evidence suggests that the Lynx family may also impact nicotinic function by altering assembly of receptors in the endoplasmic reticulum and additionally may impact early embryogenesis through other signaling pathway [28,29]. While many members of the Lynx family are found in the periphery, only a handful have been shown to be enriched in the mammalian brain [27,30]. For the most part, members of the Lynx family are thought to inhibit signaling through nicotinic receptors potentially by interfering with ligand binding [31,32], however, at least one member, Lypd6, acts to potentiate calcium currents through nAChRs [33]. This differential activity with respect to nAChR signaling could provide a means for tight regulation of nicotinic tone in specific populations of neurons. This tight regulation of nicotinic tone may be important for a number of brain functions and indeed Lynx family members have been associated with regulation of plasticity in visual cortex, learning and memory functions, sensorimotor gating and anxiety related behavior [32-36]. The importance of the Lynx family for cognitive function is further highlighted by severe intellectual disabilities displayed by patients with copy number variants (CNVs) in Lynx family loci [37]. Alternations in the expression of Lynx family members are also associated with some mouse models of psychiatric and neurodegenerative disorders [27]. Taken together, these findings suggest a critical role of the Lynx family in controlling nicotinic tone for optimal cognitive function. However, little remains known about the precise expression of Lynx family members within the brain and specifically within interneuronal populations.

Here we sought to determine the specific expression of Lynx family members with respect to GABAergic interneuron populations. We utilize open access expression data and double fluorescence *in situ* hybridization (DISH) to screen members of the Lynx family for enrichment in interneuron populations in mouse primary visual cortex (V1), a model system where the role of Lynx1 in cortical plasticity was previously established. We further characterize the GABAergic expression of two members of the Lynx family, Lynx1 and Lypd6, finding that they are expressed in discrete interneuron subpopulations. This discrete interneuronal expression of two Lynx family members makes them ideal candidates to provide tight regulation upon the convergence of GABAergic and nicotinic signaling.

## Results

### GABAergic interneurons express a subset of Lynx family members, Lynx1 and Lypd6

**Figure 1:**
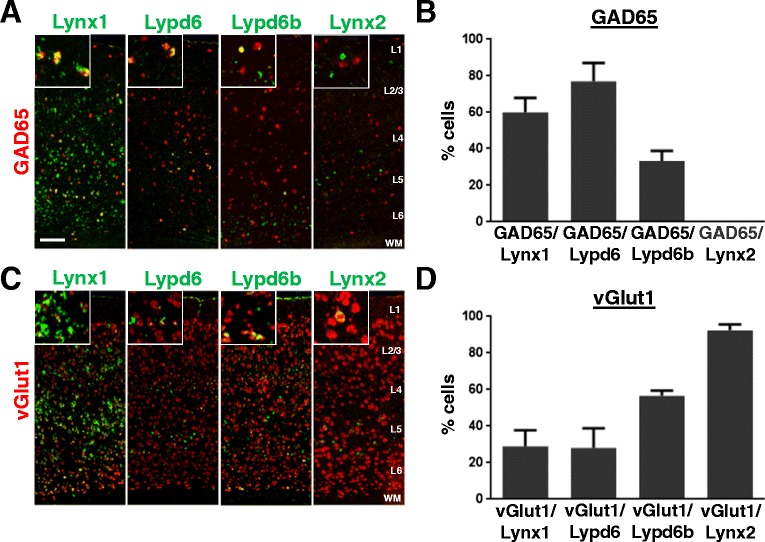
**Lynx family expression in GABAergic and glutamatergic populations in cortex. A)** Representative images from coronal sections of mouse primary visual cortex (V1) of double *in situ* hybridization labeling mRNA of GAD65 (red) and Lynx family members (green). Insets show double labeling. **B)** Quantification of the percentage of cells expressing each Lynx family member that co-express GAD65. **C)** Double in situ hybridization labeling mRNA of vGlut1 (red) and Lynx family members (green). Insets show double labeling. **D)** Quantification of the percentage of cells expressing each Lynx family member that co-express vGlut1. Error bars represent S.E.M. from n = 3-4 mice. Scale bar = 100 μm.

We first sought to elucidate members of the Lynx family that are expressed in GABAergic interneurons in mouse V1. By utilizing the Allen Brain Atlas [38], we screened for expression patterns of Lynx family members that show a scattered expression mimicking the distribution of GABAergic interneurons in the cortex and narrowed them down to six members of the Lynx family expressed in V1; Lynx1, Lypd6, Lypd6b, Lynx2, Ly6E and Ly6H. Next, we determined the differential expression pattern of these family members in GABAergic inhibitory neurons and excitatory neurons in V1. We performed DISH of each of the Lynx family members along with GAD65 or vGlut1, mutually exclusive markers of GABAergic and glutamatergic neurons respectively (Additional file 1: Figure S1). Lynx1 is expressed throughout V1 with the notable exception of layer 1. Deep cortical layers show robust Lynx1 labeling in V1. Co-labeling of Lynx1 and GAD65 mRNA revealed that Lynx1 is enriched in interneurons (60 ± 7.86% of Lynx1+ cells express GAD65) but is also expressed in a subpopulation of glutamatergic neurons (28.933 ± 8.66% of Lynx1+ cells express vGlut1) in V1 (Figure 1). The laminar expression of Lypd6 is far more restricted to layer V/VI in V1. Similarly to Lynx1, Lypd6 mRNA is enriched in GABAergic populations with 78% of Lypd6+ neurons expressing interneuron marker GAD65. As is the case with Lynx1, Lypd6 mRNA is found in a subset of glutamatergic neurons (28 ± 10.80% of Lypd6+ cells express vGlut1) that make up the minority of the Lypd6+ population (Figure 1). Lypd6b expression was sparse and restricted to deep layers in adult V1. When co-labeled with GAD65, Lypd6b showed more restricted interneuronal expression (33.33 ± 5.43% of Lypd6b + cells express GAD65). Instead, Lypd6b was primarily expressed in layer 6 glutamatergic neurons as evidenced by a high degree of overlap with vGlut1 (56.52 ± 2.886% of Lypd6b + cells express vGlut1) (Figure 1). We did not detect any expression of Lynx2 mRNA in GAD65+ neurons. Nearly all Lynx2+ cells in V1 co-localized with vGlut1 (92.45%) suggesting that the expression is nearly exclusive to excitatory neuronal populations (Figure 1). Finally, two additional Lynx family members, Ly6E and Ly6H, showed robust V1 expression but, similarly to Lynx2, were restricted to glutamatergic populations showing no detectable overlap with GAD65 expression in V1 (Additional file 1: Figure S2). Overall, the enrichment of Lynx1 and Lypd6 in cortical GABAergic populations suggests that these two nicotinic modulators may be well positioned to regulate nAChR signaling in GABAergic interneurons.

### 5HT_3A_R interneurons do not show detectable levels of Lynx1 and Lypd6

**Figure 2:**
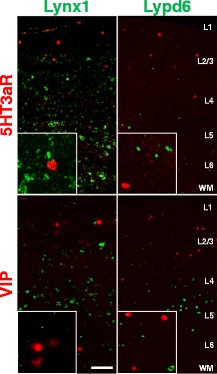
**5HT**_**3A**_ **R interneurons do not show detectable expression Lynx1 or Lypd6.** Representative images of double *in situ* hybridization labeling mRNA for 5HT_3A_R (red, top panels) or vasoactive intestinal peptide (VIP)(red, bottom panels) with Lynx1 or Lypd6 (green). Note that none of the cells labeled with either Lynx family mRNA co-localize with 5HT_3A_R or VIP positive neurons as is shown in the insets. Scale bar = 100 μm.

As both Lynx1 and Lypd6 were not expressed in all GAD65 positive GABAergic interneurons but instead smaller subpopulations (Lynx1 in 39.13% of GAD + cells, Lypd6 in 8.91% of GAD + cells: Additional file 1: Figure S3), we next aimed to identify the specific subpopulation of GABAergic interneurons expressing Lynx1 and Lypd6. Recent work established that GABAergic populations could be further subclassified through the mutually exclusive expression of one of three molecular hallmarks; the calcium binding proteins Parvalbumin (PV) and Somatostatin (SST) as well as serotonin receptor 3a (5HT_3A_R), which collectively cover 100% of GABAergic interneurons [11]. We first examined whether Lynx1 and/or Lypd6 is expressed in 5HT_3A_R + neurons in V1. 5HT_3A_R + neurons are enriched in layer1 and highly responsive to nicotine. DISH for Lynx1 and 5HT_3A_R or vasoactive intestinal peptide (VIP), a marker for a subclass of nicotinic responsive 5HT_3A_R neurons, showed no detectable levels of Lynx1 co-localized with either marker (Figure 2). Likewise, detectable levels of Lypd6 were not found to be expressed on any 5HT_3A_R + or VIP + neurons (Figure 2). These results do not rule out the possibility of subthreshold levels of Lynx family members expressed on 5HT_3A_R + neurons, however, the physiological function of any subthreshold expression in these cells is likely to be more limited.

### Mutually exclusive expression of Lynx1 and Lypd6 in PV and SST interneurons

**Figure 3:**
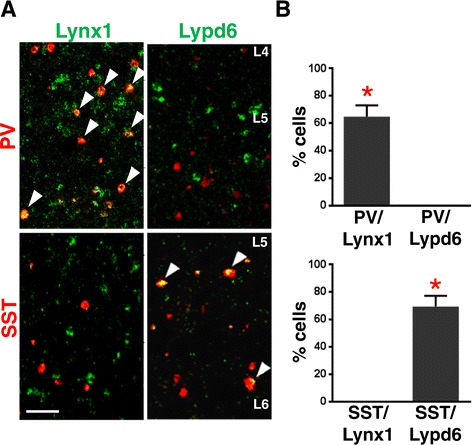
**Parvalbumin and somatostatin interneurons express distinct Lynx family members. A)** Representative images of visual cortex sections stained via double *in situ* hybridization with probes directed against markers of two interneuronal subtypes, PV (red, top panels) and SST (red, bottom panels), along with either Lynx1 (green, left panels) or Lypd6 (green, right panels). White arrowheads represent cells that show co-localization of PV or SST with either Lynx or Lypd6. **B)** Quantification of the percentage of Lynx1+ (top) or Lypd6+ (bottom) cells that co-localize with either PV or SST. *p <0.05 students *t*-test. Error bars represent S.E.M. of n = 3-4 mice. Scale bar = 50 μm.

The negative expression of both Lynx1 and Lypd6 in 5HT_3A_R + neurons indicates that they must be expressed on interneurons encompassing the other subclasses, PV and SST. Therefore, we sought to determine the expression of Lynx1 and Lypd6 in these subpopulations. We first investigated the expression of Lynx1 and Lypd6 in PV + interneurons. DISH for PV and Lynx1 confirmed the previous reports indicating nearly 90% of PV + interneurons express Lynx1 [36]. In contrast, DISH for PV and Lypd6 revealed no expression of Lypd6 in PV-cells (Additional file 1: Figure S4). Within the Lynx1+ population, approximately 60% expressed PV (Figure 3B). Therefore, restricted PV expression is consistent with the finding that ~60% of Lynx1 neurons co-express GAD65 (Figure 1).

Next, to examine the expression of Lynx1 and Lypd6 in SST + interneurons, DISH for SST and Lynx1 or Lypd6 were performed. In stark contrast to PV-cells, SST + cells showed no expression of Lynx1, but did express Lypd6. Nearly 70% of Lypd6 expressing neurons in V1 were found to express SST (Figure 3A,B bottom). Again, this is consistent with Lypd6 expression being restricted to the SST subtype of interneurons as 77% of all Lypd6 expressing neurons express GAD65 (Figure 1).

The divergent and restricted expression of Lynx1 and Lypd6 within GABAergic populations suggests a complex regulation of nicotinic signaling. Within the local cortical circuit, Lynx1 and Lypd6 each regulate nAchRs in a distinctive subtype of interneurons that may confer separate functions or could converge on a unique circuit for highly ordered control of nicotinic signaling.

### Lypd6 is enriched in subsets of SST cortical interneurons located in layer 5/6

**Figure 4:**
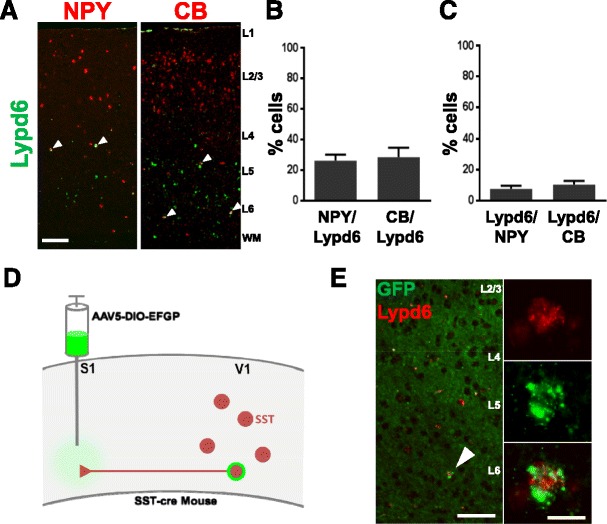
**Lypd6 is expressed in a unique subpopulation of somatostatin interneurons. A)** Representative images from double *in situ* hybridization of coronal sections of primary visual cortex labeled with probes directed against Lypd6 (green) and molecular markers of somatostatin subtypes neuropeptide Y or calbindin (red). **B)** Quantification of the percentage of total Lypd6+ neurons that co-express either neuropeptide Y or calbindin. **C)** Quantification of the percentage of each SST subtype marker (NPY or CB) that co-express Lypd6. **D)** Schematic representation of viral labeling of long-range SST neurons. **E)** Representative image of DISH for GFP and Lypd6 mRNA in V1 of SST-cre mice injected with retrograde cre-dependent GFP in S1. Arrowhead shows double labeled long-range SST neuron. Inset represents high-magnification (63×) image of double labeling. Low and high magnification = 100 μm and 15 μm respectively.

Unlike the expression of Lynx1 in nearly all PV neurons, Lypd6 expression is rather restricted to a subset of deep-layer SST neurons in V1 comprising 19.7 ± 1.7% of the total SST population (Additional file 1: Figure S5). As SST neurons can be further molecularly subclassified using the expression of calbindin (CB) and Neuropeptide Y (NPY) [39], we sought to determine if Lypd6 expression was restricted to a unique molecularly defined subtype of SST neurons. Lypd6 is expressed in both CB and NPY expressing interneuron populations (Figure 4A,B). However, the co-expression with these markers accounts for less than 60% of total Lypd6 expression suggesting that Lypd6 is also expressed in SST cells that do not contain either CB or NPY (Figure 4B). Furthermore, Lypd6 expression is restricted to only a subset of each of these populations (Figure 4C). Taken together, these results suggest that Lypd6 does not localize discretely to a single currently recognized subtype of SST neuron but could potentially identify a unique molecular subtype of deep layer SST neurons.

In general inhibitory neurons are thought to contribute mainly to local circuit activity. However, GABAergic projection neurons have been identified from a number of structures (for review [40]). In the murine neocortex, long-range cortico-cortical GABAergic neurons have been identified and shown to express SST and NPY. A large number of these inhibitory projection neurons arise from deep cortical layers [41]. To examine the expression of Lypd6 on long-range SST cortico-cortical neurons, we combined genetic and viral approaches with DISH *in vivo*. Specifically, we utilized SST-cre mice and cre-dependent retrograde viral GFP labeling using AAV5-DIO-EGFP [42] and performed DISH with probes designed to recognize Lypd6 and GFP mRNA. Virus was injected into somatosensory cortex (S1) in order to retrogradely label SST neurons projecting from V1 to S1 where long-range SST neurons have been previously reported (Figure 4D) [41]. Retrograde GFP labeling of SST neurons was sparse in V1 and mainly localized to deep cortical layers. The GFP labeled SST projection neurons only represented a small minority of the total V1 SST population. However, it is reasonable to postulate that V1 SST neurons project to regions other than S1 thus the total population of long-range SST neurons is likely underestimated by simple S1 retrograde labeling. Intriguingly, we find that a subset of the cortico-cortical SST neurons co-localize with Lypd6 (Figure 4E). With respect to the Lypd6+ population, only a small percentage expressed GFP and thus represent V1 to S1 projection interneurons. Again, it is possible that Lypd6 expressing cortico-cortical SST neurons project to other regions as well and may represent a larger total proportion than in the present experiment. These findings represent the first evidence of a potential involvement of nicotinic signaling in cortico-cortical inhibitory neurons and opens the door to new research into the functional role of SST long-range circuits and their modulation by nicotinic signaling.

## Discussion

In the present study we screened members of the Lynx family of nicotinic receptor modulators for GABAergic expression using *in situ* hybridization. We found that two members of the Lynx family, Lynx1 and Lypd6, were highly enriched in GABAergic interneuronal populations in visual cortex. Intriguingly, within GABAergic subtypes, Lynx1 is only expressed on PV + neurons while Lypd6 is restricted to SST + interneurons. Further, a subset of Lypd6 expressing neurons was found to be cortico-cortical long-range interneurons through retrograde viral labeling coupled with *in situ* hybridization.

One of the major findings of this study is the specific expression of Lynx1 in most PV interneurons but not in other subtypes of interneurons. Lynx1 has been shown to bind to nAchRs such as the homomeric α7 subunit and heteromeric α4β2 containing receptors to decrease the response to acetylcholine [31]. The response of cortical fast-spiking, putative PV interneurons, to acetylcholine could be blocked by addition of MLA, an α7 antagonist, or through genetic ablation of the α7 subunit but were unaffected by the non-α7 antagonist, DHβE [26]. Additionally, α7-null mice show decreased cortical levels of PV [43]. Taken together, these results suggest a role for homomeric α7 nAchRs in cortical PV neurons and potential modulation of these receptors by Lynx1 in PV interneurons. One potential functional implication of this modulation is the regulation of cortical plasticity. Utilizing ocular dominance (OD) plasticity in the visual cortex as a model, it was shown that Lynx1 acts as a brake molecule, limiting plasticity in adult V1 via increased expression [36]. The control of Lynx family expression appears to be complex and has not been fully elucidated, however, expression has been shown to be altered following genetic manipulations [44], in response to cholinergic perturbation [45,46] and under sensory conditions [35]. Our results showing an enrichment of Lynx1 specifically in PV neurons, but not other GABAergic subtypes, in V1 suggests that the function of Lynx1 on cortical plasticity could be at least in part mediated through PV neurons. PV neurons have been implicated in OD plasticity as the disruption of perineuronal nets surrounding PV neurons can activate OD plasticity in adults [47]. To this point, a recent study showed that pharmacogenetic inhibition of PV neurons for 1 day following monocular deprivation could reactivate OD plasticity in the adult [48]. This suggests that PV neurons play an instructive role in OD plasticity and that Lynx1 could act through modulation of nAChRs in PV neurons to suppress OD plasticity in adulthood when Lynx1 expression is elevated.

Additionally, Lynx1 could play a role in governing the synchronized network oscillations in the brain that underlie many cognitive functions. PV interneurons are thought to play a role in controlling the synchrony of network gamma oscillations in the brain. The activity of PV + basket cells is strongly coupled with gamma network oscillations in the hippocampus [49] and optogenetic disruption of PV cell activity can abolish gamma rhythm activity in cortical circuits [50]. Interestingly, α7 nAChRs have also been implicated in the control of gamma oscillations [51]. Network gamma oscillations have been strongly correlated with cognitive activity including working memory [52,53] and sensorimotor gating both of which represent common endophenotypes of psychiatric disorders including schizophrenia [31,51,52]. Both PV dysfunction in schizophrenia [54] as well as microdeletion of human 15q13.3, which includes the loss of the α7 nAChR gene and is linked with cognitive disorders such as schizophrenia, autism and mental retardation [55], have been associated with underlying disturbance of gamma oscillations. Indeed, mice with genetic ablation of α7 nAChR present with PV deficits [43] and mice genetically modified to mimic the human 15q13.3 microdeletion show dysfunctional gamma oscillatory activity [56] suggesting a potential convergence of α7 nAChR and PV centered mechanisms in cognitive function and psychiatric disorders where Lynx1 is well positioned to mediate these functions. Of note, a mouse model of 22q11 microledetion syndrome, a genetic lesion associated with increased risk of ADHD, autism spectrum disorders, schizophrenia, and other psychiatric conditions, showed significantly reduced Lynx1 expression in prefrontal cortex [57].

In addition to Lynx1 expression on PV + interneurons, we find that a separate member of the Lynx family, Lypd6 is enriched in a population of deep layer SST interneurons without expression on any other GABAergic subtype. Most cortical SST interneurons are Martinotti cells which send their axons into superficial layers where they form dense axon collateral networks in layer I [58]. Little is known about the nicotinic responsiveness of specific cortical SST subpopulations, however, Martinotti cells are morphologically and physiologically similar to oriens-lacunosum moleculare (O-LM) neurons of the hippocampus [59] that have been shown to be highly responsive to nicotinic signaling [60,61] and also densely express Lypd6 (Additional file 1: Figure S5). Nicotinic activation of O-LM neurons in CA1 facilitates long term potentiation through increases in calcium influx [61,62]. The activation or potentiation of O-LM neurons as well as neurons in the central amygdala is required for certain forms of experience dependent fear learning [63,64]. Lypd6 is an ideal candidate to mediate or modulate these activities through its ability to directly potentiate calcium currents through nicotinic receptors [33]. In cortical circuits, little is known about the involvement of SST neurons in experience-dependent plasticity but their positioning within cortical circuits make them an intriguing candidate to govern OD plasticity where the expression of Lypd6 could modulate any impact that they have. SST neurons highly innervate local PV cells placing them in an ideal position to drive the inhibition instructive to plasticity [48,65-67]. Additionally, SST neurons may directly mediate plasticity mechanisms through the inhibition of incoming information to the distal dendrites of pyramidal neurons [68,69]. These observations highlight the importance of future work designed to elucidate the role of SST neurons in OD plasticity and how these functions may be mediated by molecular modulators such as Lypd6.

Somatostatin interneurons, much like PV neurons, play a pivotal role in shaping rhythmic oscillatory activity. However, SST cells fire rhythmically in the theta frequency to help shape cortical theta rhythms [15]. Theta rhythms are linked to spatial exploration, REM sleep, memory and information packaging [70]. Patients with Schizophrenia have been reported to have a decrease in SST neurons and mRNA [71,72] as well as an increase in rhythmic theta activity [73]. Nicotine can actively induce rhythmic theta activity in the hippocampus through a GABA mediated mechanism suggesting a convergence of the two systems where Lypd6 may act in a modulatory role to gate the formation of rhythmic activity [74]. This could also help to explain the impact of Lypd6 over-expression on cognitive processes as well as the cognitive deficits in patients with CNVs in the Lypd6 locus [33,37]. We further find that a subset of long-range cortico-cortical SST neurons projecting from visual to somatosensory cortex express Lypd6. While the presence of long-range SST neurons in cortex has been previously reported [41], our study is the first to provide evidence that these neurons may be modulated by nicotinic signaling. While the long-range circuit in cortex has not been well established, it is believed that cortico-cortical SST neurons preferentially target glutamatergic neurons [75]. The functional significance of these circuits has also remained elusive. However, due to the role of GABAergic interneurons as pace-makers of rhythmic activity, it has been postulated that long-range GABAergic neurons may serve to control the interregional synchronization of network frequencies and that deficiencies in these circuits could contribute to psychiatric dysfunction [40,76].

## Conclusion

The convergence of GABAergic and nicotinic signaling plays a pivotal role in brain plasticity and cognitive functions, and dysregulation of this convergence could contribute to psychiatric conditions. Here we present evidence that some members of the Lynx family of nicotinic modulators are expressed preferentially on interneurons where they could be integral to regulating this convergence. Further, the expression of the Lynx family members on distinct interneuron subpopulations could confer intricate specificity to nicotinic regulation. This has potential significance for understanding the circuit basis of cognitive functions and for developing strategies designed to specifically target interneuron subpopulations that could produce therapeutics for psychiatric conditions that function more specifically and avoid adverse effects often associated with neuropsychiatric drugs. Direct examination of expression profiles of Lynx family members in patient samples of addictive and psychiatric disorders will be also an important area of future exploration.

## Methods

### Animals & brain processing

C57Bl/6 or somatostatin-ires-cre (SST-cre: Jackson laboratory #013044) mice were group housed under a standard 12 hr light:dark cycle (lights on at 7:00 AM:lights off at 7:00 PM) with constant temperature (23°C) and ad libitum access to food and water. All animal protocols were approved by IACUC at Icahn School of Medicine at Mount Sinai. For *in situ* hybridization experiments, mice were anesthetized with isofluorane and cervically dislocated. The brain was removed under RNAse free conditions and briefly washed *x*2 in sterile 0.9% NaCl. Subsequently, the brain was quickly frozen in a standard mold with TissueTek O.C.T. (Sakura Finetek, Torrance, CA) in a chamber of 2-methylbutane on dry ice. After freezing, brains were transferred to −80°C until sectioning. Sectioning was performed on a Leica CM3050 S cryostat (Leica Microsystems, Buffalo Grove, IL) at a thickness of 7 μm and sections were immediately placed onto Tissue Path Superfrost Gold Plus slides (Fisher Scientific, Waltham, MA). Sections were allowed to dry and then stored in slide boxes at −80°C.

### Probe making

RNA probes for GAD65, PV, SST, CB were generated using plasmids containing cDNAs gifted by Dr. Takashi Kitsukawa (Osaka University). Cloning for developing other RNA probes for *in situ* hybridization was performed in a PBluescript II SK (−) vector using a PCR based isothermal DNA assembly method. Briefly, primers were designed to linearize the vector and create non-overlapping overhang sequences. A second set of primers was utilized to amplify the gene of interest from cDNA derived from mouse cortex and create overhangs that were antisense to the vector overhang sequences. Assembly was achieved using Gibson Assembly master mix (New England Biolabs) and the resulting vector was used to transform NEB-5 (New England Biolabs) competent cells. Positive (white) colonies were picked and cultured for miniprep. All miniprep DNA was subjected to restriction digest using xba1 and xho1 enzymes (New England Biolabs) and examined for insert by gel electrophoresis. Properly inserted colonies were then amplified by 50 ml culture and subsequent midiprep using a Hispeed plasmid Midi kit (Qiagen). Plasmid DNA was then linearized using xho1 restriction digest and purified via phenol-chloroform and ethanol precipitation. Probes were synthesized using T3 RNA polymerase (Roche) and labeled with either fluorescein or digoxigenin (Roche) for double *in situ* hybridization.

Primers used to for cloning include:

- Lynx1 forward CCGCTCGAGATCCTGTTACCCTGCGTGTG
- Lynx1 reverse CGGGATCCGCTTCCTCACATCCCACAG
- Lynx2 forward CCGCTCGAGAAGGGAGTCTTTTTGTTCCCTC
- Lynx2 reverse CCGCTCGAGCCGACTGCCACTGTTCTAC
- Lypd6 forward CCGCTCGAGTGACCATGGGAAGTTATCTGTG
- Lypd6 reverse CGGGATCCAAGTCAGGCCTAGAGGTTTTCC
- Lypd6b forward: CCGCTCGAGCCTGCTTTCTCCAACTCTGACT
- Lypd6b reverse: GCTCTAGATGTTTCTGTGCTTTACATCGC
- Ly6H forward: GGTATCGATAAGCTTGATATCCTCTTTCAGGCCCTATCG
- Ly6H reverse: CCCCGGGCTGCAGGAATACGTCGACTTTTAAGATCCC
- Ly6E forward: GGTATCGATAAGCTTGATATCCTGGGCATGGAGCAAGTT
- Ly6E reverse: CCCCGGGCTGCAGGAATGCCTTCATCTGGAGGGG
- PBluescript II SK (−) forward: ATTCCTGCAGCCCGGGG
- PBluescript II SK (−) reverse: GATATCAAGCTTATCGATACC
- vGlut1 forward: TGCACAGCCACCATGGAGTT
- vGlut1 reverse: ATGATGGCATAGACGGGCAT
- 5HT_3A_R forward: GGTATCGATAAGCTTGATATCATGCGGCTCTGCATCCC
- 5HT_3A_R reverse: CCCCGGGCTGCAGGAT TCAAGAATAATGCCAAATGGA
- VIP forward: GGTATCGATAAGCTTGATATCCCTTCCCTAGAGCAGAACT
- VIP Reverse: CCCCGGGCTGCAGGAATACATCAATTTTCCTCGATTGC
- NPY forward: GGTATCGATAAGCTTGATATCTCACAGAGGCACCCAGAG
- NPY reverse: CCCCGGGCTGCAGGATAATGGGGCGGAGTCCAG
- GFP forward: GGTATCGATAAGCTTGATATCGTGAGCAAGGGCGAGGA
- GFP reverse: CCCCGGGCTGCAGGAATCAGCTCGTCCATGCCGA

### Double fluorescence *in situ* hybridization

Frozen sections were thawed and fixed in 4% paraformaldehyde. Following fixation slides were washed in PBS and incubated for 10 minutes in acetylation buffer (0.2% HCl, 1.5% Triethanolamine, 0.28% acetic anhydride). Slide were again washed in PBS and then acclimated to hybridization buffer (50% formamide, 5× SSC, 2.5% yeast tRNA, 5% Salmon sperm DNA, 10% denhardt’s solution) in a humidity chamber. Following acclimation, the buffer was replaced with new hybridization buffer containing RNA probes and incubated overnight at 72°C in the humidity chamber. The next day, slides were washed 3 × 30 minutes in 0.2× SSC at 72°C and blocked in milk solution (TBS with 0.05% Tween 20 and 1% milk). Slides were then incubated with milk containing anti-fluorescein POD antibodies (1:2000) for 2 hours at room temperature. Following incubation, slides were washed and subjected to TSA Plus DNP signal amplification (Perkin Elmer) and again washed. Next, slides were again blocked with milk solution and incubated in milk solution containing anti-DIG-akaline phosphatase and anti-DNP-KLH-488 (both 1:1000, Roche) for 2 hours at room temperature. Following incubation, slides were washed and acclimated to TBS pH 8.0 and then incubated in fast red solution (Roche) for 1 hour at room temperature. Finally, slides were washed in water and coverslipped using CC mount solution (Sigma Aldrich).

### Image and statistical analyses

Imaging was performed using either an AX10 axiophot or LSM780 confocal microscope (Zeiss). Total cell numbers were calculated for each channel using ImageJ and setting a threshold equal to 3 times the SD of the total cortex mean intensity and performing a loose size exclusion (10–100 μm). For the quantification of co-localized cells, a color-based threshold was applied using WCIF ImageJ (Wright Cell Imaging Facility) to eliminate pure green and red signal and a subsequent threshold and size exclusion was performed. To determine the percentage of co-localization, the total number of cells in a single channel was divided by the number of co-localized cells. This analysis was done for 3–4 images/animal from 3–4 separate mice. The averages are taken from the average co-localization of each mouse and presented as mean ± SEM. Where indicated, student’s *t*-test was performed using Graphpad Prism (Graphpad) software to determine statistical significance.

### Viral injection

Somatostatin-cre mice were isoflurane anesthetized and head-fixed in a mouse steortaxic apparatus (Narishige). A mid-line incision was made in the scalp and a micro-drill was used to drill a small hole in the skull over the somatosensory cortex (S1). Two injections of AAV5-syn-DIO-EGFP 500 μl each were made (from Bregma AP: 2.0, ML: 2.75, DV: 0.45; AP: 1.5, ML: 2.75, DV: 0.45) using a 2.5 μl Hamilton syringe and microinjector set to inject at 200 nl/minute. The syringe was left in place for 1 minute following the injection to reduce backflow of virus. Mice were sutured and allowed to recover from anesthesia in an empty cage over a warming pad. Following recovery, mice were returned to their home cage and allowed to maintain for 4 weeks to allow for viral expression before euthanasia for *in situ* hybridization.

## Additional file

Additional file 1: Figure S1. GAD65 and vGlut1 probes label mutually exclusive populations in V1. A representative image of a coronal section through mouse primary visual cortex showing GAD65 (red) and vGlut1 (green) mRNA labeling using double *in situ* hybridization. Scale bar = 100 μm. **Figure S2.** GABAergic interneurons do not show detectable Ly6E or Ly6H. Representative images of coronal sections through primary visual cortex showing double *in situ* hybridization labeling of vGlut1 (top panels) and GAD65 (bottom panels) mRNA with mRNA for Lynx family members Ly6E (left panels) and Ly6H (right panels). Note that neither Ly6E or Ly6H co-localize with GAD65. Scale bar = 100 μm. **Figure S3.** Only a subpopulation of GABAergic interneurons express Lynx1 and Lypd6. Quantification from double *in situ* hybridization of the percentage of GAD65+ interneurons that co-express either Lynx1 or Lypd6. Error bars represent S.E.M. of n = 3-4 mice. **Figure S4.** The majority of PV + neurons express Lynx1 but only a subpopulation of SST + neurons express Lypd6. Representative images showing mRNA double labeling of PV (top) and SST (bottom) along with either Lynx1 (left) or Lypd6 (right). Quantification of the percentage sof PV + interneurons that co-express Lynx1and Lypd6 (top graph) or SST + interneurons that co-express Lynx1 and Lypd6 (bottom graph). Error bars represent S.E.M. of n = 3-4 mice. Scale bar = 100 μm. **Figure S5.** Somatostatin positive Oriens-Lacunosum Moleculare neurons in CA1 express Lypd6. Representative images from coronal sections of DISH with probes directed against somatostatin (red) and Lypd6 (green) mRNA. Note the high overlap between somatostatin O-LM neurons and Lypd6. Scale bar = 100 μm.

## Abbreviations

GABA: Gamma-aminobutyric acid
PV: Parvalbumin
SST: Somatostatin
5HT_3A_R: Serotonin receptor 3A
nAChR: Nicotinic acetylcholine receptor
CNV: Copy number variant
DISH: Double fluorescent *in situ* hybridization
V1: Primary visual cortex
GAD65: Glutamate decarboxylase 65
vGlut1: Vesicular glutamate transporter 1
VIP: Vasoactive intestinal peptide
CB: Calbindin
NPY: Neuropeptide Y
GFP: Green fluorescent protein
S1: Primary somatosensory cortex
MLA: Methyllycaconitine
DHβE: Dihydro-β-erythroidine hydrobromide
OD: Ocular dominance
O-LM: Oriens-lacunosum moleculare
