# Supplementary material for: Cortical parvalbumin and somatostatin GABA neurons express distinct endogenous modulators of nicotinic acetylcholine receptors
Source: Mol Brain. 2014 Oct 31;7:75. doi: 10.1186/s13041-014-0075-9 (PMC4228157; doi:10.1186/s13041-014-0075-9)
Supplement: Additional file 1: Figure S1. — GAD65 and vGlut1 probes label mutually exclusive populations in V1. A representative image of a coronal section through mouse primary visual cortex showing GAD65 (red) and vGlut1 (green) mRNA labeling using double in situ hybridization. Scale bar = 100 μm. Figure S2. GABAergic interneurons do not show detectable Ly6E or Ly6H. Representative images of coronal sections through primary visual cortex showing double in situ hybridization labeling of vGlut1 (top panels) and GAD65 (bottom panels) mRNA with mRNA for Lynx family members Ly6E (left panels) and Ly6H (right panels). Note that neither Ly6E or Ly6H co-localize with GAD65. Scale bar = 100 μm. Figure S3. Only a subpopulation of GABAergic interneurons express Lynx1 and Lypd6. Quantification from double in situ hybridization of the percentage of GAD65+ interneurons that co-express either Lynx1 or Lypd6. Error bars represent S.E.M. of n = 3-4 mice. Figure S4. The majority of PV + neurons express Lynx1 but only a subpopulation of SST + neurons express Lypd6. Representative images showing mRNA double labeling of PV (top) and SST (bottom) along with either Lynx1 (left) or Lypd6 (right). Quantification of the percentage sof PV + interneurons that co-express Lynx1and Lypd6 (top graph) or SST + interneurons that co-express Lynx1 and Lypd6 (bottom graph). Error bars represent S.E.M. of n = 3-4 mice. Scale bar = 100 μm. Figure S5. Somatostatin positive Oriens-Lacunosum Moleculare neurons in CA1 express Lypd6. Representative images from coronal sections of DISH with probes directed against somatostatin (red) and Lypd6 (green) mRNA. Note the high overlap between somatostatin O-LM neurons and Lypd6. Scale bar = 100 μm. [file 13041_2014_75_MOESM1_ESM.pdf]

Additional File 1.

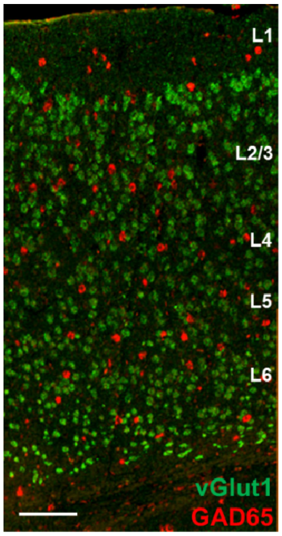

GAD65 and vGlut1 probes label mutually exclusive populations in V1. A representative image of a coronal section through mouse primary visual cortex showing GAD65 (red) and vGlut1 (green) mRNA labeling using double *in situ* hybridization. Scale bar = 100µm.

Additonal File 2

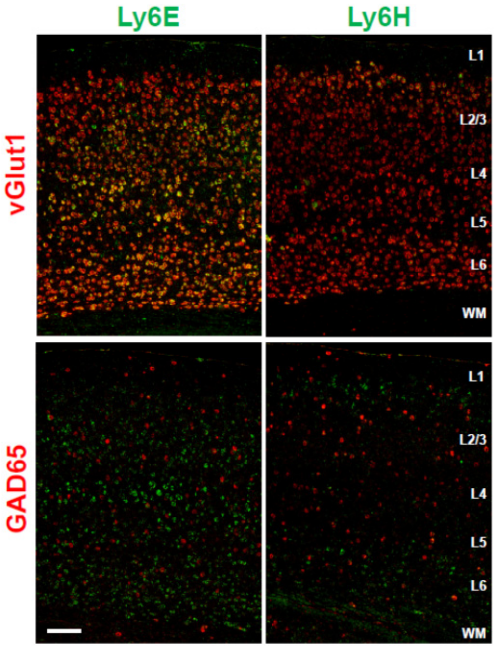

GABAergic interneurons do not show detectable Ly6E or Ly6H. Representative images of coronal sections through primary visual cortex showing double *in situ* hybridization labeling of vGlut1 (top panels) and GAD65 (bottom panels) mRNA with mRNA for Lynx family members Ly6E (left panels) and Ly6H (right panels). Note that neither Ly6E or Ly6H co-localize with GAD65. Scale bar = 100µm.

Additional File 3.

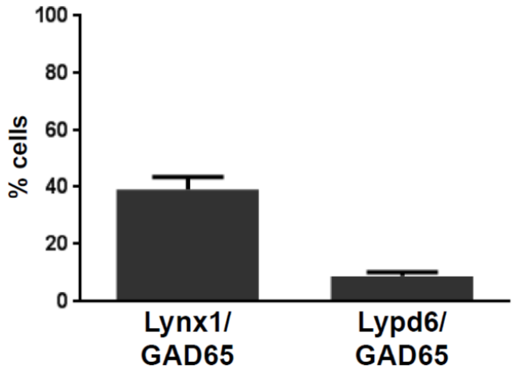

Only a subpopulation of GABAergic interneurons express Lynx1 and Lypd6. Quantification from double *in situ* hybridization of the percentage of GAD65+ interneurons that co-express either Lynx1 or Lypd6. Error bars represent S.E.M. of n = 3-4 mice.

Additional File 4.

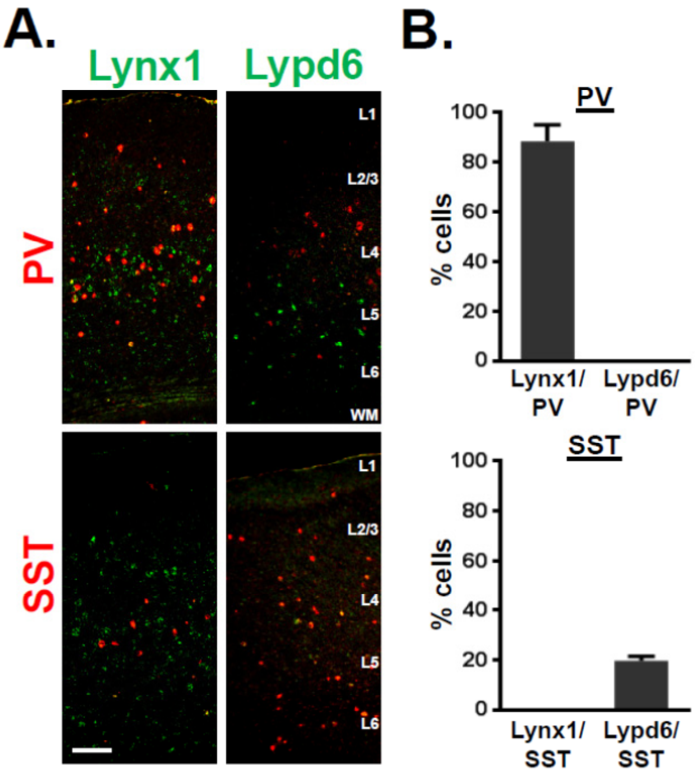

The majority of PV+ neurons express Lynx1 but only a subpopulation of SST+ neurons express Lypd6. Representative images showing mRNA double labeling of PV (top) and SST (bottom) along with either Lynx1 (left) or Lypd6 (right). Quantification of the percentage of PV+ interneurons that co-express Lynx1and Lypd6 (top graph) or SST+ interneurons that co-express Lynx1 and Lypd6 (bottom graph). Error bars represent S.E.M. of n =3-4 mice. Scale bar = 100µm

Additional File 5.

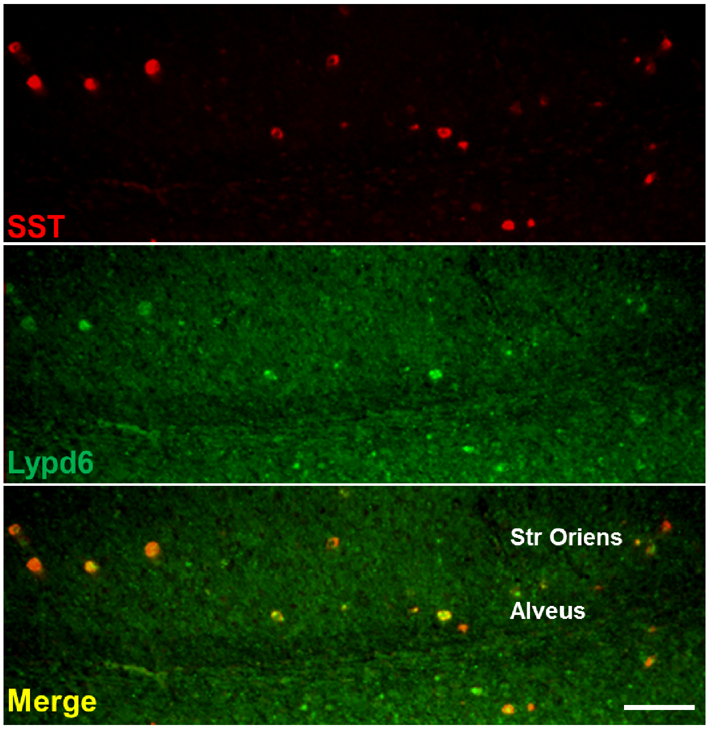

Somatostatin positive Oriens-Lacunosum Moleculare neurons in CA1 express Lypd6.
Representative images from coronal sections of DISH with probes directed against somatostatin (red) and Lypd6 (green) mRNA. Note the high overlap between somatostatin O-LM neurons and Lypd6. Scale bar =100µm.
